# The Role of Bromodomain Proteins in Regulating Gene Expression

**DOI:** 10.3390/genes3020320

**Published:** 2012-05-29

**Authors:** Gabrielle A. Josling, Shamista A. Selvarajah, Michaela Petter, Michael F. Duffy

**Affiliations:** Department of Medicine, The Royal Melbourne Hospital, The University of Melbourne, Australia; E-Mails: gjosling@student.unimelb.edu.au (G.A.J.); s.selvarajah@student.unimelb.edu.au (S.A.S.); mpetter@unimelb.edu.au (M.P.)

**Keywords:** bromodomain, histone acetylation, histone modifications, histone code, epigenetics

## Abstract

Histone modifications are important in regulating gene expression in eukaryotes. Of the numerous histone modifications which have been identified, acetylation is one of the best characterised and is generally associated with active genes. Histone acetylation can directly affect chromatin structure by neutralising charges on the histone tail, and can also function as a binding site for proteins which can directly or indirectly regulate transcription. Bromodomains specifically bind to acetylated lysine residues on histone tails, and bromodomain proteins play an important role in anchoring the complexes of which they are a part to acetylated chromatin. Bromodomain proteins are involved in a diverse range of functions, such as acetylating histones, remodeling chromatin, and recruiting other factors necessary for transcription. These proteins thus play a critical role in the regulation of transcription.

## 1. Introduction

The post-translational modification of histones plays an important role in regulating gene expression in eukaryotes. Each nucleosome consists of approximately 147 base pairs of DNA wrapped around an octamer containing two of each of the core histones H2A, H2B, H3, and H4, or their variants. Each of the histones contains a well-conserved histone fold domain as well as an unstructured amino-terminal tail [1]. These histone tails can be modified in a variety of ways, including through methylation, acetylation and phosphorylation. Although most modifications are found on the amino-terminal tails of histones, histones can also be modified on their carboxyl terminal ends. These modifications are thought to influence processes such as transcription in two main ways: firstly, by changing the physical interaction between histones and DNA to alter the chromatin structure, and secondly, by acting as a binding surface for the recruitment of other proteins which can then directly or indirectly influence transcription.

Histone lysine acetylation is one of the best studied histone modifications and is generally associated with active genes. Histones are acetylated by enzymes called histone acetyltransferases (HATs), and deacetylated by histone deacetylases (HDACs). Reversal of histone acetylation and deacetylation occurs rapidly, suggesting that histone acetylation is extremely dynamic and thus allows for precise control of transcription [2]. 

This review will discuss the role that histone acetylation plays in gene regulation and the mechanisms by which it influences transcription, with a particular focus on *trans* factors recruited to acetylated histones through bromodomains.

## 2. Histone Acetylation and the Regulation of Transcription

Although histone acetylation has been demonstrated to play a role in the regulation of processes such as replication, nucleosome assembly, and DNA repair [3,4,5], its best characterised role is in the regulation of transcription [6]. Many histone lysine acetylations have been identified and characterised in different organisms.

Many studies have mapped various histone acetylations using chromatin immunoprecipitation (ChIP). In humans and mice H3K9/14ac (histone 3 acetylated at lysines 9 and 14) is enriched at transcriptional start sites [7], and in *Drosophila melanogaster* H3ac and H4ac are both associated with active genes [8]. In budding yeast, genome-wide ChIP shows that H3K9ac, H3K14ac, and H4K5,8,12,16ac are found close to the transcriptional start sites of genes and generally their presence correlates positively with transcription [9], although H4K16ac also directly recruits the HDAC Sir2 which then removes H4K16ac and propagates silent heterochromatin [10]. 

In the most comprehensive study of histone acetylations, 18 different histone acetylations on all the core histones were mapped in human CD4^+^ T cells using ChIP-seq [11]. The patterns of enrichment seen for well-characterised modifications such as H3K9ac were similar to those seen in other organisms, suggesting that at least some histone acetylations may be conserved in function. However, as many of the acetylations examined have not yet been characterised in other organisms, it is possible that some of these patterns are specific to CD4^+^ T cells. Although all the modifications examined positively correlated with gene expression, a number of different patterns of enrichment were observed. All the acetylations were enriched close to transcriptional start sites, but many were also found in other positions. Whereas H3K9ac is highly enriched at the transcriptional start site of active genes and absent throughout the coding sequence, some modifications like H2AK5ac, H3K14ac, and H4K12ac are also present throughout the coding regions of highly expressed genes. In addition, many modifications such as H3K18ac and H3K27ac are enriched in enhancers, consistent with other observations of H3K27ac [12,13]. The different patterns of enrichment of various histone acetylations suggests that they are involved in different aspects of transcriptional regulation; for instance, modifications found near transcriptional start sites are likely to be involved in the initiation of transcription, whereas those found in coding sequences may play a role in elongation.

Histone variants are also often subject to histone modifications. H2A.Z and H3.3 are well-characterised variants of the canonical histones H2A and H3 respectively. The incorporation of both of these histone variants together into a nucleosome makes it less stable, and this is generally associated with active transcription [14]. Acetylation of histone variants plays an important role in gene regulation. For example, acetylated H2A.Z is enriched in highly transcribed genes, whereas unacetylated H2A.Z is found in silent genes in chickens [15], humans [16] and yeast [17]. In *Tetrahymena*, acetylation of H2A.Z is essential for viability and leads to a more open chromatin structure [18]. Interestingly, in yeast H2A.Z acts as a barrier to block the spread of heterochromatin, however it was found that unacetylatable H2A.Z was incapable of blocking this spread [19]. In mammalian cells and *Drosophila melanogaster* H3.3 is more abundantly acetylated than H3, though the role that these modifications play in regulating gene expression remains unclear [20,21].

Histone acetylation is thought to affect transcription through two main mechanisms: firstly, by altering the interaction between DNA and histones leading to changes in chromatin structure, and secondly, by recruiting proteins. It has long been known that histone acetylation increases the accessibility of chromatin for transcription factors [22] and also that it inhibits the formation of tightly packaged chromatin and consequently leads to higher levels of transcription *in vitro* [23]. It is hypothesised that this is caused by the neutralisation of the positive charge on the lysine residue, thus reducing the affinity of histones for the negatively charged DNA. This then results in a loosening of chromatin structure, allowing for greater access by the transcriptional machinery [24]. Acetylation of H4 and in particular H4K16 seems to be especially important in destabilising chromatin folding [25,26,27]. *In vitro*, H4K16ac inhibits the formation of the higher order 30 nm fibres, presumably due to the neutralisation of the positive charge on this residue [28]. 

As well as directly affecting chromatin structure, histone acetylation can influence transcription through the recruitment of proteins. For example, H3K9ac and H3K14ac are necessary for the recruitment of the general transcription factor TFIID to the promoter of the IFN-β gene in humans [29]. Using *in vitro* nucleosome disassembly and transcription assays, H3K14ac has also been shown to be necessary for nucleosome disassembly at promoters by the histone chaperone Nap1 which leads to transcription [30]. These examples indicate that histone acetylation does have a functional link to gene expression, although for some genes H3K9ac is not essential for transcription despite being correlated with it. This is based on the observation that deletion of the HAT responsible for acetylating H3K9 does not affect expression of at least some target genes in mouse embryonic fibroblasts, although this did result in the absence of H3K9ac at these loci [31].

In addition, perturbing the normal patterns of histone acetylation by mutation of HATs and HDACs leads to changes in gene expression [32,33,34]. Deleting HDACs in yeast results in a general increase in the expression of genes where the HDACs normally act, which is consistent with the positive correlation between histone acetylation and transcription [35]. Mutating acetylatable residues in histones also leads to changes in gene expression. Dion *et al*. mutated all four acetylatable residues in H4 in all possible combinations in budding yeast and examined changes in gene expression by microarray. Interestingly, whereas acetylations of lysines 5, 8, and 12 had a cumulative effect on expression of the same genes, loss of H4K16ac affected expression of a distinct set of genes. These results indicate that histone acetylation may affect gene expression through more than one pathway [36].

## 3. Recruitment of *Trans* Factors to Acetylated Histones by Bromodomains

Although histone acetylation was initially thought to primarily affect gene expression through its direct effect on the histone-DNA interaction, it also plays an important role in recruiting proteins which can themselves influence transcription and other chromatin-templated processes. It has been hypothesised that histone modifications constitute a “histone code”, in which different patterns of histone modifications are “read” by various proteins to produce an effect on gene expression [37,38]. These proteins have specific domains which recognise particular modifications [39]. For example, acetylated histones are typically recognised by the bromodomain, whereas methylated histones are recognised by various domains, including the chromodomain and PHD finger domain. 

The structures of many bromodomains have now been solved. These include the bromodomains of the HATs PCAF [40,41], TAFII250 [42], CBP [43], and yeast and human orthologues of GCN5 [44,45], as well as those of the transcriptional co-activators Brd2 [46] and Brd4 [47], and the chromatin remodeling component Brg1 [48]. Recently the structures of 29 human bromodomains were solved, greatly expanding the number of available bromodomain structures [49]. Despite the functional diversity of these proteins, the structure of the bromodomain itself is well conserved. The bromodomain consists of a left-handed bundle of four alpha helices (α_A_, α_B_, α_C_, and α_Z_). Two loops formed between the α_B_ and α_c_ helices and the α_Z_ and α_A_ helices (BC and ZA, respectively) form a hydrophobic pocket, which is where the protein interacts with the acetylated lysine residue. Despite the structural similarity between bromodomains, the overall sequence is not highly conserved beyond the residues which are directly involved in acetyl-lysine binding [50]. These differences in sequence may account for the differences in binding specificities seen between bromodomains [49]. A single bromodomain frequently displays affinity for multiple acetylated residues, often on different histones [51,52,53].

Many proteins contain more than one bromodomain, and these often have different binding affinities [49,52]. This may increase the overall binding affinity of the protein for acetylated histones; for example, the tandem bromodomain protein TAFII250 binds strongly (K_d_ 5 µM) to the H4K5,8,12,16ac peptide compared to a singly acetylated H4 peptide (K_d_ 39 µM) {Jacobson, 2000 #528}. It is also of note that many bromodomain proteins contain additional domains [54]. The bromodomain is often found in combination with the PHD finger domain, which generally recognises methylated lysine residues in histone tails, but the bromodomain is also found in combination with other histone-binding domains such as the MBT domain and the WD40 domain. This potentially allows these proteins to recognise multiple modifications through different domains, and is consistent with the “histone code” hypothesis. For example, the NURF chromatin remodeling complex subunit BPTF contains both a bromodomain and a PHD finger, and has been shown to bind to both H4K16ac and H3K4me3 in the same nucleosome [55]. 

Interestingly, some bromodomains are able to bind to two acetylated residues simultaneously [49,56]. Although Brdt and Brd4 both contain two bromodomains, in both cases the first bromodomain can bind to diacetylated H4 and the crystal structure shows that the bromodomain contacts both acetylated residues [49,56]. The bromodomains of Brd2 and Brd3 also bind with greater affinity to acetylated histone peptides than to the single acetylated peptide, suggesting that they may also be able to bind two acetylated residues simultaneously [49]. This provides another possible mechanism for reading multiple histone modifications.

**Figure 1:**
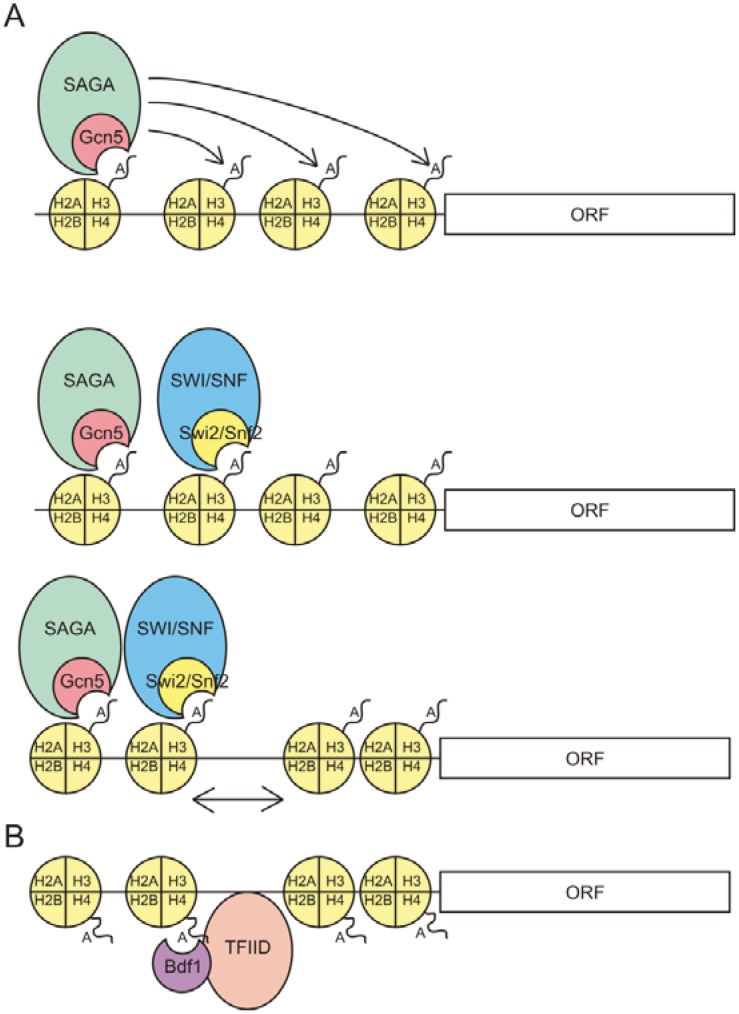
Examples of mechanisms by which bromodomain proteins regulate gene expression in budding yeast. (**a**) The bromodomain of Gcn5 binds to acetylated H3 and anchors the histone acetyltransferase complex Spt-Ada-Gcn5 acetyltransferase(SAGA) to nucleosomes, allowing SAGA to acetylate adjacent nucleosomes. The chromatin remodeling complex SWI/SNF then binds to the acetylated nucleosomes and remodels the promoter region to allow for formation of the pre-initation complex and ultimately transcription; (**b**) The bromodomain-extraterminal (BET) bromodomain protein Bdf1 binds to acetylated H4 and recruits the general transcription factor TFIID.

Consistent with their binding to acetylated histones, bromodomain proteins often play a role in gene activation. Many bromodomain proteins are part of a larger complex where they act by anchoring the complex to acetylated chromatin. Bromodomain proteins are involved in many different stages in transcriptional regulation, as the examples shown in Figure 1 indicate. Most bromodomain proteins fall into one of three categories: components of histone acetyltransferase complexes, components of chromatin remodeling complexes, and bromodomain-extraterminal (BET) proteins. Several of these will be discussed below, and these are summarised in Table 1.

**Table 1:** Selected bromodomain proteins in budding yeast and mammals.

Protein	Organism	Complex	Histone-binding specificity	Other interactions	Function
**Components of histone acetyltransferases:**								
Gcn5	Yeast	SAGA, SALSA/SLIK, ADA	H3ac, H4K16ac [45,53]		Required for acetylation of nucleosomal histones leading to gene activation [57,58,59,60,61,62,63]
Spt7	Yeast	SAGA	H3K9ac [53]		
p300/CBP	Mammals		p300: H3ac [64], H1K74ac, H2AK5ac, H2AK15ac, H2AK36ac, H2BK43ac, H2BK46ac, H3K56ac, H3K79ac, H3K115ac, H3K122ac, H4K5ac, H4K44ac [49] CBP: H2AK15ac, H3K56ac [49], H4K20ac [65]	Acetylated p53 [43], RNA polymerase II [66], TBP [67], TFIIB [68], RNA helicase A [69], c-Jun [70], c-Fos [71]	Acetylates histones [72,73], general and specific transcription factors [74,75]; recruitment of transcriptional machinery leading to transcription initiation [76,77]
**Components of chromatin remodeling complexes:**								
Swi2/Snf2	Yeast	SWI/SNF	H3ac, especially H3K14ac [52]		Catalytic component. Remodels chromatin in promoters and also evicts nucleosomes in elongation thus enhancing transcriptional elongation [78,79,80]
Rsc1	Yeast	RSC	H3ac (weakly) [52]		
Rsc2	Yeast	RSC	H3ac (weakly) [52]		
Rsc4	Yeast	RSC	H2Bac and H3ac, especially H3K14ac [52,81]		
Sth1	Yeast	RSC	H3K14ac, H3K115ac, H2AK21ac [52]		Catalytic component. Remodels chromatin in promoters and plays a role in enhancing elongation [82,83,84]
**BET bromodomains:**								
BDF1	Yeast	SWR1	H3ac, H4ac [52,53,85,86,87]	TFIID subunit Taf67 [86]	Recruitment of TFIID thus leading to transcription initiation [88], incorporation of H2A.Z into nucleosomes [89]
BDF2	Yeast		H2Bac and H3ac [52]	TFIID subunit Taf67 [86]	Partially redundant with Bdf1 [88]
Brd2	Mammals		H4K12ac, H4K5/8ac [46,47,90,91,92], H1K74ac [49]	E2F transcription factors [93], TBP [94], unknown HAT [95], TAFII250, components of SWI/SNF complex [96]	Increases transcription of E2F- regulated genes [93]
Brd4	Mammals		H3ac, H4ac [47,51,97], H2AK5ac, H2AK36ac, H2AK75ac, H2BK43ac, H3K18ac, H3K36ac, H3K37ac, H3K56ac, H4K5ac, H4K20ac, H4K44ac [49]	Mediator complex [98,99], P-TEFb complex [100,101]	Increases transcription by RNA polymerase II [100,101]

### 3.1. Histone Acetyltransferase Complexes

One of the main classes of bromodomain proteins are components of histone acetyltransferase complexes. Many histone acetyltransferase complexes contain a component with a bromodomain, although that protein may not itself have HAT activity. The bromodomain is generally responsible for anchoring the HAT complex to acetylated chromatin, allowing it to acetylate adjacent nucleosomes. The presence of the bromodomain in the HAT complex presumably allows for spreading of histone acetylation as it allows HATs to be recruited to nucleosomes that are already acetylated. Some HATs also play an additional role in recruiting specific and general transcription factors.

In yeast, the protein Gcn5 is a component of the SAGA (Spt-Ada-Gcn5 acetyltransferase) complex which positively regulates transcription by RNA polymerase II and is enriched in promoters of active genes [57]. Gcn5 has two mammalian orthologues: GCN5 and PCAF. Gcn5 itself contains a bromodomain and also a HAT domain. The bromodomain of Gcn5 binds mostly to acetylated H3 and to a lesser extent to acetylated H4, though it is able to bind to H4K16ac [45,53]. Whereas recombinant Gcn5 acetylates H3K14 only, in the context of the SAGA complex it is able to acetylate other residues of H3 as well [102], suggesting that other components of the complex are important in determining the specificity of HAT activity. The bromodomain of Gcn5 is not required for HAT activity *in vitro*, though cells in which Gcn5 has been knocked out are not fully complemented by Gcn5 lacking a bromodomain, indicating that the bromodomain of Gcn5 does play a functional role *in vivo* [59,60].SAGA complexes containing Gcn5 lacking a bromodomain have a reduced ability to acetylate nucleosomal histones but not free histones [61]. Gcn5 lacking a bromodomain is still recruited to nucleosomes by the activator GAL4-VP16, but unlike the wildtype protein is not retained when the activator is removed [62]. These data suggest an important role for the bromodomain of Gcn5 in anchoring the SAGA complex to nucleosomes and allowing it to acetylate histones.

Gcn5 is also a component of the HAT complexes ADA and SALSA/SLIK [58,63]. Although these complexes have some components in common with SAGA, they also contain other subunits [61,63,103]. Less is known about the functions of SALSA/SLIK and ADA than about those of SAGA, but mutation of unique subunits in each complex produces distinct phenotypes [63]. This suggests that these complexes have at least some different functions. Although the HAT activities of SALSA/SLIK and SAGA have similar substrate specificity against H3K9, 14, 18, and 23 [63], ADA acetylates only H3K14 and H3K18, again indicating that Gcn5 does not solely determine which residues these complexes acetylate [58].

Another component of the SAGA complex, Spt7, also has a bromodomain. Spt7 is thought to be important for the assembly and integrity of the SAGA complex [104]. A truncated form of Spt7 is also present in the SALSA/SLIK complex [105]. Although Spt7 knockout cells display growth and transcriptional defects, deletion of the bromodomain alone results in no mutant phenotype [106]. In mutants expressing Spt7 lacking its bromodomain, no change in SAGA recruitment or retention is seen compared to wildtype cells [62]. The recombinant bromodomain of Spt7 is able to bind H3K9ac [53], but binding apparently does not occur in the context of SAGA given that deletion of the bromodomain has no effect. When the bromodomain of Gcn5 is replaced with that of Spt7, the SAGA complex binds as well to nucleosome arrays as when wildtype Gcn5 is present, showing that the Spt7 bromodomain is functional [62].

Although some bromodomain proteins regulate gene expression only through their HAT activity, others are also able to regulate transcription more directly through interactions with general and specific transcription factors. The mammalian proteins CBP and p300 each have a bromodomain and a HAT domain, as well as several other domains involved in protein binding. CBP and p300 belong to the same class of HATs and are very similar in structure [107]. They are both enriched in enhancers and promoters of genes, and although they have largely overlapping distributions throughout the genome there are also many loci that contain only one of them [108]. Consistent with this, CBP and p300 have some distinct functions in gene regulation [109,110]. For example, in embryonal carcinoma F9 cells, loss of p300 prevents retinoic-acid-induced differentiation whereas loss of CBP does not [109]. The bromodomain of p300 binds to all the core histones but preferentially to H3, whereas the bromodomain of CBP binds to H4K20ac [49,64,65]. A recent landmark study using SPOT blot analysis identified many additional acetylated residues bound by both proteins, many of which were confirmed using isothermal titration calorimetry [49]. CBP showed strong binding to H2AK15ac and H3K56ac, and p300 bound to H1K74ac, H2AK5ac, H2AK15ac, H2AK36ac, H2BK43ac, H2BK46ac, H3K56ac, H3K79ac, H3K115ac, H3K122ac, H4K5ac, and H4K44ac. The bromodomains are required for binding to chromatin and are important in their functions as transcriptional co-activators [64,111]. In addition, they are also able to recognise acetylated non-histone proteins, and in particular transcription factors; for example, the bromodomain of CBP can bind to acetylated p53 [43]. This shows that although bromodomain proteins generally affect gene expression through their function in anchoring their cognate complexes to acetylated chromatin, they can also act by binding to non-histone proteins. 

In addition to having HAT activity against all core histones [72,73], CBP and p300 are also able to acetylate non-histone substrates such as the general transcription factors TFIIE and TFIIF and the specific transcription factor GATA1 [74,75]. In addition to their bromodomain and HAT domain, CBP and p300 have several other domains which allow them to bind to a very large number of proteins and this interaction allows them to act as transcriptional co-activators; for example, they bind to TBP, TFIIB, RNA helicase A, and RNA polymerase II [66,67,68,69,112]. They are also able to bind to specific transcription factors such as c-Jun and c-Fos [70,71]. At the IFN-β promoter, CBP is recruited by an enhanceosome and is then in turn able to recruit the RNA polymerase II holoenzyme which leads to transcription [76,77]. Thus, CBP and p300 regulate gene expression not just by acetylating histones, but also by recognising non-histone proteins, acetylating non-histone substrates, and recruiting RNA polymerase II.

### 3.2. Chromatin Remodeling Complexes

The second major class of bromodomain proteins is those found in ATP-dependent chromatin remodeling complexes. These complexes utilise the energy generated from ATP hydrolysis to alter the contacts between DNA and histones, allowing for the movement of nucleosomes. The bromodomain components of chromatin remodeling complexes are critical for their recruitment to the genes that they regulate. Subsequent chromatin remodeling can then influence gene expression by mechanisms such as improving access to the promoter region for the transcriptional machinery and allowing for transcriptional elongation in coding regions.

The chromatin remodeling SWI/SNF complex regulates a subset of genes in budding yeast [34], and organisms such as *Drosophila* [113] and mammals [114,115,116] also have complexes that contain subunits homologous to the yeast SWI/SNF subunits. The complex is found in the promoter region of these genes and its chromatin remodeling activities allow for greater access by the transcriptional machinery [78,79]. Additionally, the complex remains associated with RNA polymerase II during elongation and is involved in histone eviction [80]. There is evidence that it can also play a role in repressing transcription [34,117]. Swi2/Snf2 is the catalytic ATP-dependent helicase component of the SWI/SNF complex and it contains a bromodomain which preferentially binds to acetylated H3 and in particular H3K14ac [52,118]. It is homologous to the human proteins BRG1 and hBRM [119]. *In vitro* assays have shown that stable binding of the SWI/SNF complex requires acetylation of histones by HAT complexes, indicating that the bromodomain is involved in anchoring the complex to acetylated histones [79]. Furthermore, deletion of the bromodomain of Swi2/Snf2 results in reduced binding to acetylated histones and subsequently reduced chromatin remodeling [120]. Acetylation of H3 increases bromodomain-dependent mobilisation of nucleosomes as well as H2A/H2B dimer displacement which is consistent with acetylated H3 recruiting the chromatin remodeling function of SWI/SNF through the Swi2/Snf2 bromodomain [118]. 

The SWI/SNF and SAGA complexes interact with a similar set of gene-specific transcriptional activators, and genes whose expression is dependent on the SWI/SNF complex are also dependent on the SAGA complex [121,122]. It has been shown that the SAGA subunit Gcn5 is essential for the recruitment of the SWI/SNF complex and for stabilising this complex for chromatin remodeling and subsequent transcriptional activation [123]. Acetylation of H3 by the SAGA complex also precedes recruitment of the SWI/SNF complex at the human IFN-β promoter [29]. The recruitment of SWI/SNF by SAGA-mediated H3 acetylation is summarised in Figure 1A. At the yeast *HO* promoter, however, recruitment of the SWI/SNF complex is required for subsequent recruitment of the SAGA complex [124]. This suggests that although the recruitment of the SWI/SNF complex to chromatin is generally dependent on the presence of SAGA, this order of recruitment may be gene dependent. Interestingly, SAGA is also able to inhibit SWI/SNF by acetylating Swi2/Snf2, which results in reduced binding of the complex to acetylated histones [125].

The yeast RSC complex shares two identical subunits with the SWI/SNF complex, and the two complexes also contain several subunits with a high degree of homology including the catalytic subunit [126]. As is the case for the SWI/SNF complex, acetylation of H3 recruits the yeast RSC complex in a bromodomain-dependent manner which in turn facilitates nucleosome mobilization [118]. The *Drosophila* BAF and mammalian BAP complexes are evolutionary counterparts of the yeast RSC complex [113,127]. The RSC complex is enriched in many class II and class III promoters [82], and loss of RSC complex function results in changes in nucleosome occupancy at promoters and reduced transcription [83]. It also plays a role in transcriptional elongation [84]. Although the RSC complex is far more abundant and regulates a different set of genes to the yeast SWI/SNF [117,128], they have similar biochemical properties [126]. The RSC complex contains multiple subunits which together comprise eight of the 15 bromodomains of yeast, and components of the RSC complex are essential for viability [129,130]. The bromodomain-containing components include the catalytic subunit Sth1, as well as Rsc1, Rsc2, and Rsc4 which each have two bromodomains [81,130,131]. Sth1 is homologous to the human proteins BRG1 and hBRM, while Rsc1, Rsc2 and Rsc4 have homology to BAF180 [119]. The bromodomain of Sth1 is critical for the function of the RSC complex and binds strongly to acetylated H3K14, H3K115 and H2AK21 as well as to other acetylated residues with lower affinity [52,130]. Although only one of the two bromodomains within the Rsc1 and Rsc2 proteins is vital for function, both bromodomains of Rsc4 are required [81,130,131]. The bromodomains of Rsc1 and Rsc2 bind weakly to acetylated H3, whereas the bromodomains of Rsc4 bind strongly to all the core histones but particularly H2B and H3 [52]. The second bromodomain of Rsc4 binds to H3K14ac, and this acetylation is critical for recruitment of the RSC complex [81]. 

### 3.3. BET Bromodomain Proteins

The third main class of bromodomain proteins is the bromodomain-extraterminal (BET) family. There are two BET bromodomain proteins in budding yeast (Bdf1 and Bdf2) and five in higher eukaryotes (Brd2, Brd3, Brd4, and Brdt). These proteins contain two bromodomains at their amino terminal end as well as a conserved extraterminal (ET) domain at the carboxyl end which serves as a protein-protein interaction module. Like many other bromodomain proteins, BET bromodomain proteins are primarily involved in regulating transcription through their interactions with other proteins; for example, BET bromodomain proteins are able to recruit specific and general transcription factors and some also play a role in chromatin remodeling. BET bromodomain proteins are thus involved in gene regulation via a number of different mechanisms.

Bdf1 and Bdf2 are two similar BET bromodomain proteins in budding yeast. Whereas Bdf1 binds preferentially to acetylated H3 and H4, Bdf2 binds predominantly to H2B and H3 [52,53,85,86,132]. The two bromodomains within each protein have different binding specificities [52]. Mutation of the bromodomains of Bdf1 results in a loss in histone binding and leads to downregulation of certain genes, particularly those which are TBP-associated factor (TAF) dependent [87]. Deletion of Bdf1 results in defects in growth and transcription, whereas deletion of Bdf2 results in no phenotypic change [85,86]. In wildtype cells Bdf1 and Bdf2 have different genome-wide distributions, but when Bdf1 is deleted Bdf2 replaces it in some genes suggesting that they are partially redundant in function [88]. 

Bdf1 and Bdf2 associate with the general transcription factor complex TFIID and specifically interact with the TFIID subunit Taf67 via their extraterminal domain [86]. Deletion of Bdf1 results in loss of TFIID recruitment to some genes, indicating a role for Bdf1 in the recruitment of TFIID [88]. This is shown in Figure 1B. Bdf1 is also part of the SWR1 chromatin remodeling complex [89]. The SWR1 complex is a SWI/SNF-like complex that alters chromatin structure by exchanging the canonical histone H2A for the histone variant H2A.Z in an ATP-dependent manner [133]. Although Bdf1 is not required for H2A.Z incorporation *in vitro*, deletion of Bdf1 results in greatly reduced levels of incorporated H2A.Z *in vivo* [134]. Mutation of EsaI (a component of the NuA4 HAT complex) decreases H4 acetylation and subsequent Bdf1 binding as well as binding of TFIID and SWR1 [88]. This indicates that recruitment of SWR1 and TFIID is dependent on the acetylation of H4 by NuA4 and thus suggests that Bdf1 plays a key role in recruiting these complexes.

Brd2 (also known as Fsrg1 and RING3) is a mammalian BET protein with kinase activity that is associated with the promoters of a subset of cell cycle genes [93,135]. Brd2 binds as a dimer to acetylated residues on H4; its first bromodomain binds to H4K12ac, and the second bromodomain in each of the two molecules in the dimer binds to H4K5ac and H4K8ac, respectively [46,51,90,91,92]. The first bromodomain also binds strongly to H1K74ac [49]. Like many bromodomain proteins, Brd2 functions as part of a complex. It is involved in the activation of cell cycle genes regulated by E2F transcription factors, and has been shown to form a complex with these transcription factors [93]. Brd2 is responsible for recruiting the general transcription factor TATA-binding protein (TBP) to the E2F complex, and the first bromodomain is required for the interaction of Brd2 with TBP [94]. Whereas an acetylated nucleosomal template was able to be transcribed in the presence of wildtype Brd2 *in vitro*, in the presence of Brd2 containing mutations in both bromodomains transcription did not occur [135]. This shows that the bromodomains of Brd2 play a critical role in the protein’s function in regulating transcription by allowing it to be recruited to target genes. Brd2 has been shown to be associated with an unknown histone acetyltransferase [95], RNA polymerase II [136], as well as the general transcription factor TAFII250 and components of the SWI/SNF chromatin remodeling complex [96]. It is unclear whether all of these elements are present within a single complex, or whether there are multiple Brd2-containing complexes which fulfil different functions.

Brd4 (also known as MCAP and Hunk1) has a similar structure to Brd2, but as well as having an extraterminal domain it has an additional C-terminal motif which is also involved in interacting with other proteins [137]. The ET domain of Brd4 has been shown to interact with a number of different proteins including some involved in chromatin remodeling [137]. Brd4 binds to acetylated H3 and H4 [97]. The first bromodomain binds predominantly to acetylated H3, whereas the second bromodomain has greater affinity for acetylated H4 [47]. Its first bromodomain has been shown to bind strongly to H2AK75ac and H3K56ac, and its second bromodomain binds strongly to H2AK5ac, H2AK36ac, H2BK43ac, H3K18ac, H3K36ac, H3K37ac, H3K56ac, H4K5ac, H4K20ac, and H4K44ac [49]. Brd4 is part of some forms of the Mediator co-activator complex which is necessary for the transcription of many genes through its interaction with RNA polymerase II [98,99]. It is also able to bind to the active form of the positive transcription elongation factor b (P-TEFb) complex [100,101]. P-TEFb allows for transcriptional elongation by phosphorylating RNA polymerase II [138]. Over-expression of Brd4 causes P-TEFb to increase phosphorylation of RNA polymerase II which leads to increased transcription [100], showing that Brd4 is important in regulating transcription. The acetyl-lysine binding ability of the bromodomain plays an important role in this function of Brd4, as treatment with the histone deacetylase inhibitor trichostatin increases P-TEFb recruitment [100]. Brd4 can also directly phosphorylate RNA polymerase II [139]. Recently, a role in chromatin condensation has also been suggested for Brd4 by experiments in which Brd4 was knocked down leading to chromatin decondensation [140]. Unlike other bromodomain proteins and transcription factors, BET bromodomain proteins such as Brd2 and Brd4 remain associated with mitotic chromosomes [90,97].

## 4. Histone Acetylation Pathways as Drug Targets

The important role that histone acetylation plays in gene regulation means that changes in histone acetylation are often associated with disease. For example, in promyeloctyic leukaemia a mutant form of the retinoic acid receptor associates with a histone deacetylase complex, which leads to changes in histone acetylation [141,142]. For this reason, proteins involved in histone acetylation pathways may be good drug targets. Histone deacetylase inhibitors have been successfully used as treatments for cancer, inflammation, and neurological disorders such as schizophrenia [143,144,145].

Recently, bromodomain proteins themselves have emerged as interesting new drug targets when it was demonstrated that they can be inhibited with high specificity [146]. Bromodomain proteins have been implicated in a number of diseases; for example, NUT midline carcinoma (NMC) is caused by a mutation leading to a fusion between the BET bromodomain protein Brd4 and NUT, which produces the Brd4-NUT oncoprotein [147]. Knockdown of Brd4 has been shown to lead to reduced expression of the Myc oncogene, suggesting a likely mechanism by which Brd4 plays a role in cancer [101]. A small molecule inhibitor of Brd4 called JQ1 has been developed, and treatment with JQ1 leads to growth arrest in NMC cells and has anti-tumour activity in xenograft models [148]. Treatment with JQ1 leads to a down-regulation of Myc as well as genes regulated by Myc [149]. JQ1 shows anti-tumour activity in several cancer types in which BET bromodomain proteins have been implicated [149,150,151]. Similarly, the bromodomain inhibitor I-BET151 has been shown to have efficacy in mixed lineage leukaemia in mouse and human cell lines [152]. I-BET151 and another bromodomain inhibitor, I-BET, have anti-inflammatory effects and suppress bacteria-induced sepsis and thus may make good immunomodulatory drugs [153,154]. In addition, novel bromodomain proteins in infectious agents such as malaria parasites may be good drug targets [155]. These studies show the potential of bromodomain proteins as drug targets in multiple diseases, including cancer, inflammatory and infectious diseases.

## 5. Conclusions

Histone acetylation affects gene expression both through direct physical effects on chromatin structure and through recruitment of complexes containing *trans* factors such as bromodomain proteins. HAT and chromatin remodeling complexes containing bromodomain proteins are recruited to target genes by the bromodomain binding to acetylated chromatin (Figure 1A). The complexes then facilitate transcription initiation and elongation. Bromodomain proteins also directly participate in recruiting general and specific transcription factors (Figure 1B). The many acetylations thus far described show considerable redundancy in their capacity to recruit the pool of bromodomain proteins. Many individual bromodomain proteins are present in multiple complexes, which allow for a diversity of enzymatic functions to be recruited by overlapping sets of histone acetylations. The enzymatic functions of these complexes cover a range of possible mechanisms of gene regulation. The application of next generation sequencing technology to chromatin immunoprecipitation and expression profiling is revealing the detailed associations that exist between gene expression, histone acetylations and the *trans* factors that bind them. These data are revealing many novel interactions that will provide useful targets for future therapeutic strategies.
